# Trilobatin, a Component from *Lithocarpus polystachyrus* Rehd., Increases Longevity in *C. elegans* Through Activating SKN1/SIRT3/DAF16 Signaling Pathway

**DOI:** 10.3389/fphar.2021.655045

**Published:** 2021-04-15

**Authors:** Na Li, Xi Li, Yan-Ling Shi, Jian-Mei Gao, Yu-Qi He, Fei Li, Jing-Shan Shi, Qi-Hai Gong

**Affiliations:** ^1^ Key Laboratory of Basic Pharmacology of Ministry of Education and Joint International Research Laboratory of Ethnomedicine of Ministry of Education, Zunyi Medical University, Zunyi, China; ^2^ Key Laboratory of Basic Pharmacology of Guizhou Province, Zunyi Medical University, Zunyi, Guizhou, China; ^3^ Department of Clinical Pharmacotherapeutics, School of Pharmacy, Zunyi Medical University, Zunyi, China

**Keywords:** trilobatin, anti-aging, *Caenorhabditis elegans*, SKN1, SIRT3, DAF16

## Abstract

Trilobatin (TLB) is an effective component from *Lithocarpus polystachyrus* Rehd. Our previous study revealed that TLB protected against oxidative injury in neuronal cells by AMPK/Nrf2/SIRT3 signaling pathway. However, whether TLB can delay aging remains still a mystery. Therefore, the present study was designed to investigate the possible longevity-enhancing effect of TLB, and further to explore its underlying mechanism in *Caenorhabditis elegans* (*C. elegans*). The results showed that TLB exerted beneficial effects on *C. elegans*, as evidenced by survival rate, body movement assay and pharynx-pumping assay. Furthermore, TLB not only significantly decreased ROS and MDA levels, but also increased anti-oxidant enzyme activities including CAT and SOD, as well as its subtypes SOD2 andSOD3, but not affect SOD1 activity, as evidenced by heat and oxidative stress resistance assays. Whereas, the anti-oxidative effects of TLB were almost abolished in SKN1, Sir2.3, and DAF16 mutant *C. elegans*. Moreover, TLB augmented the fluorescence intensity of DAF16: GFP, SKN1:GFP, GST4:GFP mutants, indicating that TLB increased the contents of SKN1, SIRT3 and DAF16 due to fluorescence intensity of these mutants, which were indicative of these proteins. In addition, TLB markedly increased the protein expressions of SKN1, SIRT3 and DAF16 as evidenced by ELISA assay. However, its longevity-enhancing effect were abolished in DAF16, Sir2.3, SKN1, SOD2, SOD3, and GST4 mutant *C. elegans* than those of non-TLB treated controls. In conclusion, TLB effectively prolongs lifespan of *C. elegans*, through regulating redox homeostasis, which is, at least partially, mediated by SKN1/SIRT3/DAF16 signaling pathway.

## Introduction

Aging, a sophisticated biological process, is characterized as a gradual recession in biological functions at the molecular, organelle, tissue, even the entire organism level (Liu and Li, 2020; Zhang et al., 2020). Aging is considered as a greatest risk factor for vast of the mortality and morbidity in the world, due to it results in multiple increment incidence rates of cardiovascular disease, cerebrovascular disease, cancer, and neurodegenerative disease (Majidinia et al., 2019). Since the world population is aging at an accelerated pace, aging-related disorders aggravate the burden in economic terms and the suffering in human. Emerging evidence indicates that hesitate aging per se will be potential to simultaneously delay aging-related disorders (Gomes et al., 2020). However, the detailed mechanisms of aging are complex. Emerging evidence manifests that oxidative stress and mitochondrial dysfunction play imperative roles in aging (Park et al., 2020). Concomitant reduction in antioxidant capacity and/or increase in oxidative stress, which is referred to as the imbalance between the antioxidant enzyme system and production of reactive oxygen species (ROS). ROS is termed as a heterogeneous population of biologically active intermediation, deriving from the erobic metabolism as by-products and display a dual role in biology (Zhang et al., 2019). That is, ROS exhibits beneficial role in normal physiological conditions, while, superabundant ROS is a saboteur to aging or aging-related disorders. Of note, recent researches suggest that the silent information regulator 2 (SIR2) family is a highly conserved nicotinamide adenine dinucleotide-binding catalytic domain and involves in the cellular events mediating aging-related diseases (Talarowska et al., 2019; Hallows, et al., 2011). Sirtuin 3 (SIRT3) is the vital mitochondrial protein deacetylase of SIR2 family and it is known as an emerging mediator of mitochondrial responses to oxidative stress or aging, as well as modulates cell survival (Quan et al., 2020). Thus, the SIRT3 is expected to be a promising target for prophylaxis and treatment of aging.

**FIGURE 1:**
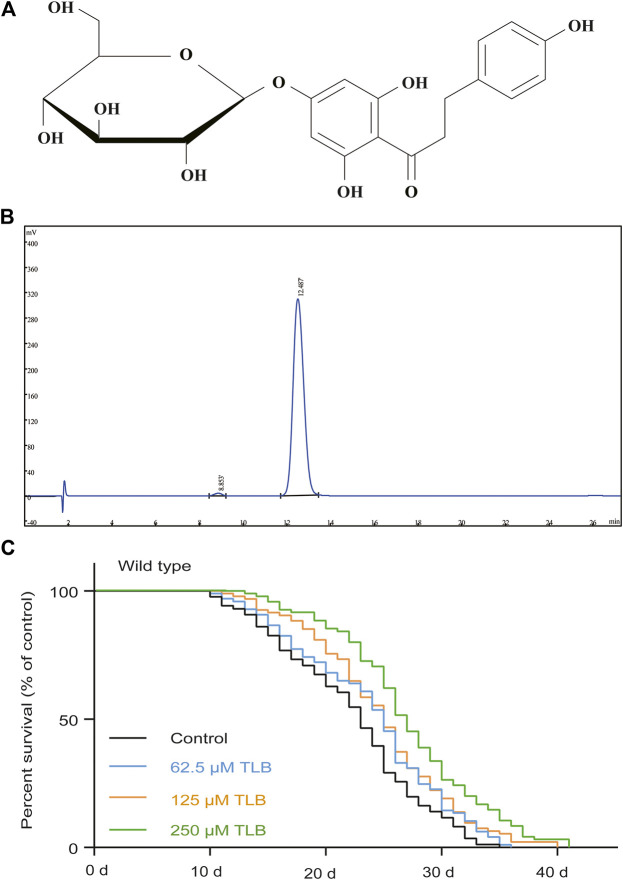
TLB extended lifespan under normal cultivate condition at 20°C in wild type *C. elegans*. **(A)** The structure of TLB. **(B)** The high-performance liquid chromatography of TLB. Wild type *C. elegans* were treated with or without 62.5, 125, and 250 μM TLB. **(C)** The Kaplan-Meier survival curves of wild type *C. elegans* (*n* = 86–97). Data were presented as mean ± SEM.

Recent reports demonstrate that traditional Chinese medicine (TCM) or folk medicines and their active compounds exhibit effective anti-aging activities, such as resveratrol and curcumin (Kumar et al., 2018; Li et al., 2018). It is interesting to note that trilobatin (Figures 1A,B), a natural bioflavonoid derived from the leaves of *Lithocarpus polystachyus* Rehd, exerts multiple curative effects such as hypoglycemic effect, antiviral effect (Lei et al., 2018; Yin et al., 2018). Intriguingly, our previous study revealed that TLB effectively protected against oxidative injury in neuronal PC12 cells through regulating mitochondrial ROS homeostasis mediated by AMPK/NRF2/SIRT3 signaling pathway (Gao et al., 2018). However, whether TLB can anti-aging remains still unknown. Therefore, the aim of this study was designed to investigate the effect of TLB on aging in *C. elegans* and further to explore its potential mechanisms.

## Materials and Methods

### Chemicals and Reagents

TLB (purity ≥98%) was purchased from Guangdong Kedi Medical Technology Corporation, and TLB was dissolved in dimethyl sulfoxide (DMSO) for storage to 1 mM and diluted in PBS. The final concentration of DMSO in the media was less than 1‰ (v/v). Agar powder (A8190), yeast extract (LP0021), Tryptone (LP0042), DMSO (D8371), cholesterol (C8280) and glycerol (G8190) were purchased from Solarbio (Beijing, China). ROS ELISA assay kit (RJ21993), MDA ELISA assay kit (RJ25505), SOD ELISA assay kit (RJ21986), SOD1 ELISA assay kit (RJ25669), SOD2 ELISA assay kit (RJ25670), SOD3 ELISA assay kit (RJ25671), glutathione (GSH) ELISA assay kit (RJ21991), CAT ELISA assay kit (RJ25506), and glutathione peroxidase (GSH-Px) ELISA assay kit (RJ25510) were purchased from Shanghai Ren Jie Biotechnology (Shanghai, China). SIRT3 ELISA kit (KD15803), FOXO3 ELISA kit (KD20035), and NRF2 ELISA kit (KD14201) were purchased from Guangzhou Kedi Biotechnology (Guangzhou, China) (-) -tetramisole hydrochloride (SLBN8309V) was purchased from Sigma-Aldrich (St Louis, MO, United States). Paraquat (FY-C10104076) was purchased from Nantong Feiyu Biotechnology (Jiangsu, China).

### *C. elegans* Culture

All strains were afforded by Caenorhabditis Genetics Center (CGC, University of Minnesota, United States). The strains were used in this experiment as follow: Bristol N2 (wild type, WT); DA1116 *eat2* (ad1116) II, RB654 *sir2.3* (ok444) X, EU1 *skn1* (zu67) IV, CF1038 *daf16* (mu86) I, CF1139 muls61 (DAF16:GFP), CL2166 dvIs19 III (GST4:GFP), KN259 huIs33 (SOD3:GFP), LD1008 ldEx9 (SKN1:GFP), and TJ375 gpIs1 (heat shock protein 16.2:GFP, HSP16.2:GFP). *C. elegans* were maintained on nematode growth medium (NGM) with *Escherichia coli* OP50 at 20°C.

### Lifespan Experiment

All age-synchronized strains were cultivated for 2-3 generations on fresh NGM plates at 20°C before used for lifespan analysis. Thereafter, late L4 larvae or young adults were shifted to 35 mm NGM plates with or without different concentrations of TLB (62.5, 125, and 250 μM) containing live or heat-inactivated *E. coli* OP50 (65°C for 1 h). The L4 *C. elegans* were defined as a start time point (d 0) for lifespan assay. *C. elegans* that failed to response to a mechanical stimulation were scored as dead when they failed to response to a mechanical stimulation (with a platinum wire (Huang et al., 2017). Since 250 μM TLB exhibited the best lifespan prolonged effect on lifespan, it was selected as the optimal concentration in following experiments.

### Heat Stress and Oxidative Stress Resistance Assays

The effects of TLB on thermo-tolerance and oxidative stress in *C. elegans* were determined using thermo-tolerance assay and oxidative stress tolerance assay, respectively. In brief, the age-synchronized late L4 larvae or young adult *C. elegans* were shifted onto plates with or without TLB (250 μM) for 72 h. Thereafter, the adult *C. elegans* were incubated at 35°C for 2 h or treated with 10 mM paraquat at 20°C for all the life of *C. elegans* to determine the effects of TLB on heat stress and oxidative stress, respectively. The survival rates of *C. elegans* were recorded once per day as previous study (Zheng et al., 2017).

### Determination of Progeny Production, Body Movement and Pharynx-Pumping

The breeding experiment was proceeded as previous study (Shen et al., 2018). In brief, *C. elegans* were cultured on plates with or without TLB (250 μM). Next, the parents of *C. elegans* were removed to fresh plates per day until reproduction ceased, and the hatched *C. elegans* were recorded each day. Moreover, body movement assay was performed as described previously (Yang et al., 2015). Briefly, age-synchronized late L4 larvae or young *C. elegans* were bred on NGM agar plates with or without 250 μM TLB at 20°C for 3, 6, and 9 days, then *C. elegans* were shifted to fresh plates. Thereafter, *C. elegans* body movement were counted with times of sinusoidal motion during 20 s. In addition, the effects of TLB on food intake in *C. elegans* were determined using counting the times of the pharynx contraction for 20 s.

### Measurement of ROS and MDA Levels

*C. elegans* were raised in the absence or presence of 250 μM TLB for 72 h as mentioned above. Then, *C. elegans* were cultivated in NGM plates containing 10 mM paraquat and *C. elegans* homogenate was measured using ROS ELISA assay and lipid peroxidation MDA ELISA assay. In brief, *C. elegans* were harvested and rinsed thrice with ddH_2_O. The total protein WT *C. elegans* or mutant *C. elegans* were extracted and centrifuged for 10 min at 10,000 × *g*, 4°C. Then levels of MDA and ROS were detected by ELISA kits under the manufacturer’s instructions.

### Measurement of Antioxidant Enzyme Activities

Briefly, *C. elegans* were treated as mentioned above. The total protein of *C. elegans* was extracted and centrifuged for 10 min at 10,000 × *g*, 4°C, then the supernatant was collected. Thereafter, activities of SOD, SOD1, SOD2, SOD3, CAT, GSH, and GSH-Px were detected using appropriate ELISA kits according to the manufacturer’s protocol.

### Measurement of Expressions of SKN1, SIRT3, and DAF16

*C. elegans* were raised in the absence or presence of 250 μM TLB for 72 h as mentioned above. The total protein of *C. elegans* was extracted and centrifuged for 10 min at 10,000 × *g*, 4°C, then the supernatant was collected. Thereafter, expressions of SKN1, SIRT3, and DAF16 were detected using appropriate ELISA kits according to the manufacturer’s protocol.

### Determination of Fluorescence in Transgenic *C. elegans*

The age-synchronized late L4 larvae or young transgenic adult *C. elegans* including *CF1139* (DAF16:GFP), *CL2166* (GST4:GFP), *KN259* (SOD3:GFP), *LD1008* (SKN1:GFP), *TJ375* (HSP16.2:GFP) were raised on NGM dishes with or without 250 μM TLB for 72 h at 20°C. Thereafter, TJ375 mutants were treated by heat stress at 35°C for 2 h, and the fluorescence of *C. elegans* were observed under a fluorescence microscopy (Olympus BX53 + DP80, Olympus, Japanese) at wavelength with excitation/emission (360/420 nm) filters after the transgenic mutants were anesthetized using (-) -tetramisole hydrochloride (5 mM). The fluorescence of transgenic *C. elegans* were quantified using the Image Pro Plus 6.0 software.

### Determination of Lipofuscin in *C. elegans*

The effects of TLB on aging in *C. elegans* were evaluated by detecting lipofuscin level. Briefly, *C. elegans* were treated with or without TLB for 5 or 10 days as mentioned above. Then *C. elegans* were randomly selected and washed with M9 buffer for three times and then anesthetized with 5 mM levamisole as described in previously study (Yang et al., 2018). The intestinal spontaneous fluorescence of *C. elegans* were observed using fluorescence microscopy (Olympus BX53 + DP80, Olympus, Japanese) at wavelength with excitation/emission (360/420 nm) filters. The fluorescence of *C. elegans* were quantified using the Image Pro Plus 6.0 software.

### Statistical Analyses

The data were confirmed and conducted by using Graphpad Prism version 6.0 (Graphpad software, Inc. San Diego, United States). All data were expressed as mean ± SEM and analyzed with the SPSS 18.0 software (SPSS, Inc. Chicago, IL, United States). Besides, the data of lifespan experiments and stress resistance experiments were performed using the Kaplan–Meier survival analysis. All results were verified by at least three independent experiments. *p* < 0.05 was considered statistically significant.

## Results

### TLB Prolonged Longevity in *C. elegans* Under Normal Cultivate Condition

In order to confirm whether TLB can prolong the lifespan, the WT *C. elegans* were treated with different concentrations (62.5, 125, and 250 μM) of TLB. The results indicated that TLB (62.5, 125, and 250 μM) markedly prolonged the mean longevity by 7.7, 11.7, and 22.1% in *C. elegans* than those of non-TLB treated controls, respectively (Figure 1B, Table 1). These findings demonstrated that TLB led to a prominent increasement of lifespan in a concentration-dependent manner.

**TABLE 1:** Effects of TLB on the mean lifespan of *C. elegans*.

Genotype	Mean lifespan ±SEM (days)		Chang (%)	Number of *C. elegans* (N)	*p* Value
	Untreated	Treated			
N2	22.2 ± 0.7	23.9 ± 0.7^a^	7.7	183	0.023
		24.9 ± 0.6^b^	11.7	180	0.009
		27.2 ± 0.6^c^	22.1	181	<0.001
N2 (oxidative stress)	9.9 ± 0.3	12.0 ± 0.4	22.1	194	<0.001
N2 (heat stress)	10.0 ± 0.5	11.5 ± 0.5	14.2	189	0.035
*skn-1* (zu67) IV	20.2 ± 0.5	20.8 ± 0.5	2.9	203	0.403
*daf-16* (mu86)	19.5 ± 0.3	20.1 ± 0.3	3.1	200	0.112
*sir-2.3* (ok444) X	19.3 ± 0.6	20.9 ± 0.7	8.2	177	0.052
*sod-3* (gk235) X	18.2 ± 0.5	17.8 ± 0.4	-−.4	172	0.437
*sod-2* (ok1030) I	19.6 ± 0.6	19.8 ± 0.5	1.3	176	0.918
*gst*-4&msp-38 (ok2358) IV	21.9 ± 0.4	20.5 ± 0.5	−6.5	178	0.120
*eat-2* (ad1116) II	22.6 ± 0.6	24.7 ± 0.6	9.3	169	0.008

^a^ Unless otherwise specified, TLB-treated concentration was 250 μM.

^b^ TLB-treated concentration was 62.5 μM.

^c^ TLB-treated concentration was 125 μM.

### TLB Increased Body Movement and Attenuated Pharynx-Pumping Rate or Lipofuscin but Not Affected the Propagation of *C. elegans*

**FIGURE 2:**
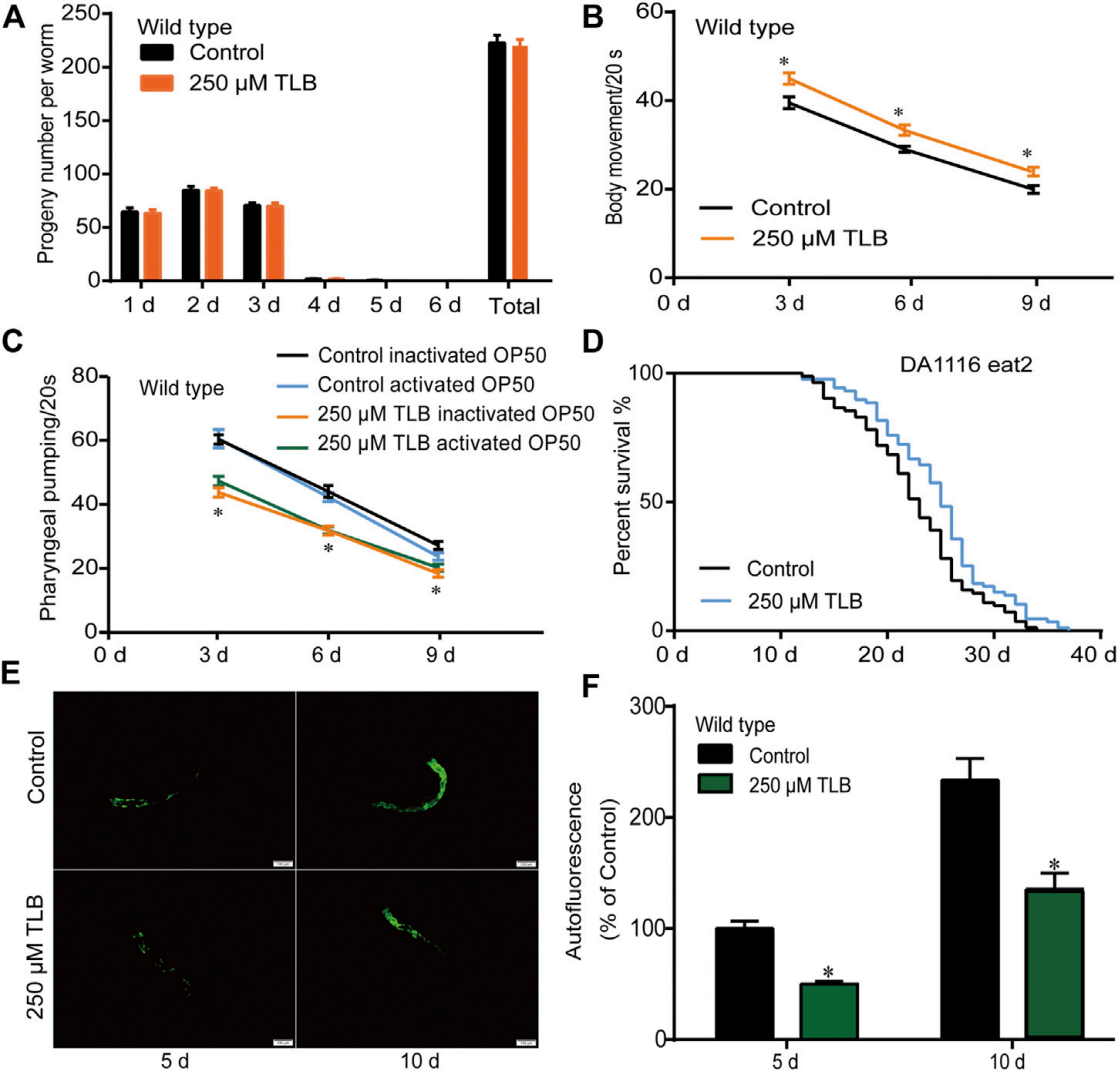
TLB changed behaviors in aging *C. elegans*. **(A)** The number of progeny per day and the total number of progeny of *C. eleganss* treated with 250 μM TLB at the onset of age-synchronized late L4 or young larvae *C. elegans* (*n* = 10). **(B)** Age-synchronized late L4 larvae or young *C. elegans* were bred on NGM agar plates with or without 250 μM TLB at 20 °C for 3, 6, and 9 days, *C. elegans* body movement were counted with times of sinusoidal motion during 20 s (*n* = 20). **(C)** The effects of TLB on food intake in *C. elegans* were determined the times of the pharynx contraction for 20 sat 20°C for 3, 6, and 9 days (*n* = 10). *p* values were calculated by independent-samples *t* test. **(D)** The survival curves of *eat2* (DA1116) *C. elegans* raised on 0 and 250 μM TLB at 20°C (*n* = 82–87). The lifespan was analyzed by the Kaplan-Meier test, and *p* values were calculated by the log-rank test. **(E)** Representive lipofuscin autofluorescence image of 0 and 250 μM TLB *C. elegans* on the 5 and 10 days of adulthood. The fluorescence of *C. elegans* were observed under a fluorescence microscopy. **(F)** The mean pixel fluorescence of transgenic *C. elegans* were quantified using the Image Pro Plus 6.0 software. Magnification × 100; scale bar, 100 μm (*n* = 20). Data were presented as mean ± SEM. ^*^ *p* < 0.05 vs. non-TLB treated controls.

The results showed that TLB did not affect the total progeny number of *C. elegans* (Figure 2A). Moreover, TLB markedly augmented the movement ratio of *C. elegans* than that of non-TLB treated controls (Figure 2B). Afterward, we detected the times of pharyngeal pumping to evaluate the food intake of *C. elegans*, the results showed that TLB markedly reduced the times of pharyngeal pumping when *C. elegans* were cultured both in live or heat-inactivated OP50 (Figure 2C), indicating that TLB reduced the decline of pharyngeal pumping rate accompanied with aging. Of note, there was no significant difference on the times of pharyngeal pumping of *C. elegans* that cultured both in live or heat-inactivated OP50, and due to it is easier to observe the change of *C. elegans* in heat-inactivated OP50 than that in live OP50, the heat-inactivated OP50 was used in the following experiment. Furthermore, *eat2 C. elegans*, an abnormal pharyngeal pumping mutant *C. elegans*, was used to confirm the attenuative effect of TLB on pharyngeal pumping. Of note, the results suggested that longevity-enhancing effects of TLB on *C. elegans* was partly abolished in *eat2* mutant *C. elegans* than that of non-TLB treated controls (Figure 2D). These findings suggested that beneficial effects of TLB on lifespan was partially dependent on food intake, and there were probably other factors that still needed further explore. Intriguingly, TLB also dramatically attenuated the endogenous lipofuscin than that of non-TLB treated controls (Figures 2E,F), which further indicated that TLB played an active role in delaying aging.

### TLB Enhanced the Lifespan in *C. elegans* Under Heat Stress Condition

**FIGURE 3:**
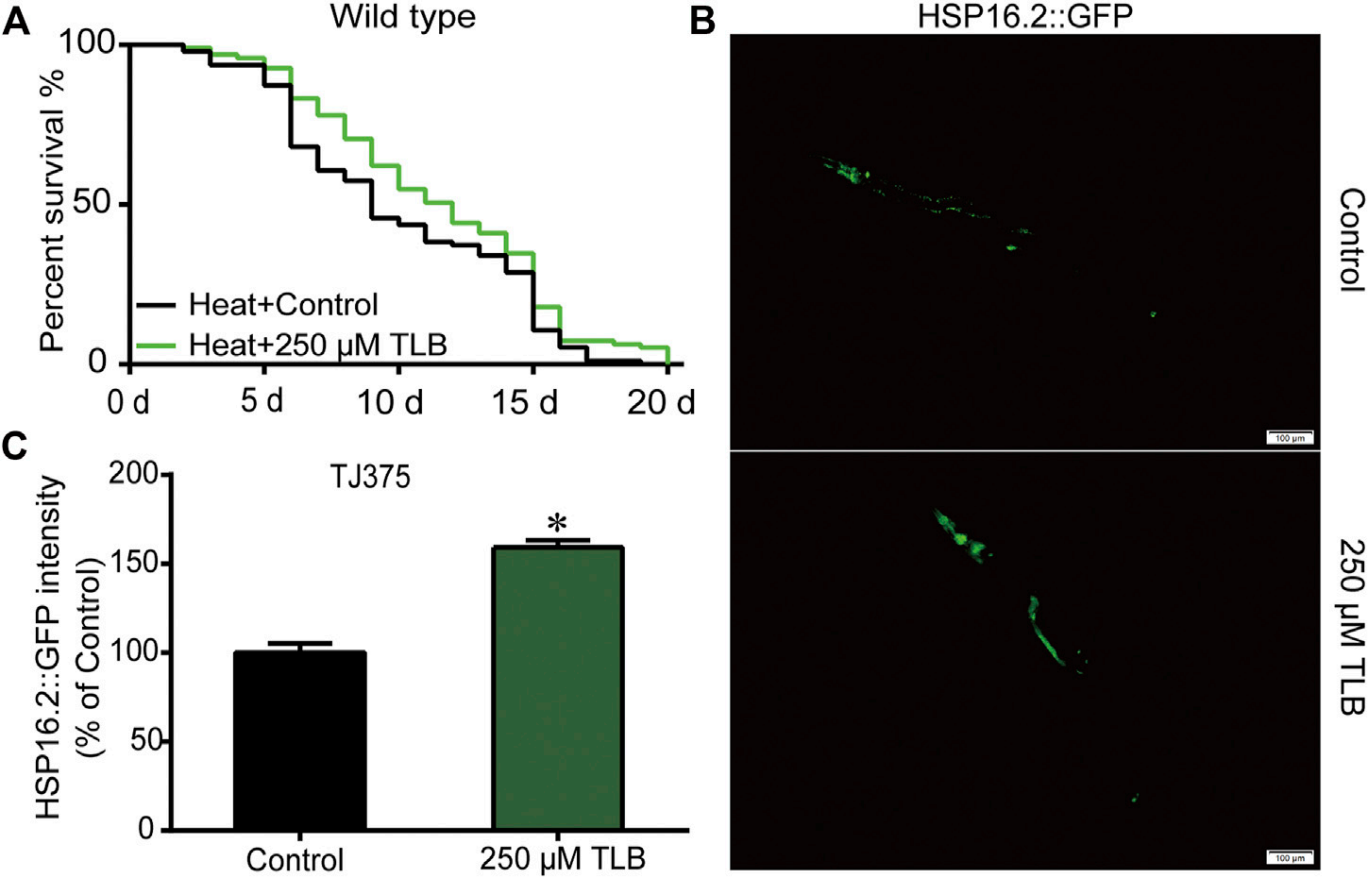
TLB reinforced the heat stress tolerance of *C. elegans*. Wild type *C. elegans* were treated with or without TLB under 35°C condition. **(A)** The Kaplan-Meier survival curves under 35°C condition (*n* = 94–95). **(B)** The HSP16.2 transgenic *C. elegans* were treated by heat stress at 35°C for 72 h, and the fluorescence of *C. elegans* were observed under a fluorescence microscopy. **(C)** The mean pixel fluorescence intensity of protein HSP16.2 were quantified using the Image Pro Plus 6.0 software (*n* = 20). Magnification × 100; scale bar, 100 μm. Data were presented as mean ± SEM. ^*^ *p* < 0.05 vs. non-TLB treated controls.

The results showed that the mean lifespan of *C. elegans* treated with TLB was markedly augmented than that of non-TLB treated controls, which manifested that TLB observably heightened *C. elegans* resistance to thermal tolerance (Figure 3A). Analogously, HSP16.2 gene levels were also measured using transgenic strain TJ375 (HSP16.2:GFP). The results revealed that the HSP16.2 levels of *C. elegans* were enhanced observably by TLB treated than that of non-TLB treated controls (Figures 3B,C), manifesting that TLB significantly increased the capacity for resistance to thermal stress.

### TLB Enhanced the Lifespan in *C. elegans* Under Oxidative Stress Condition

**FIGURE 4:**
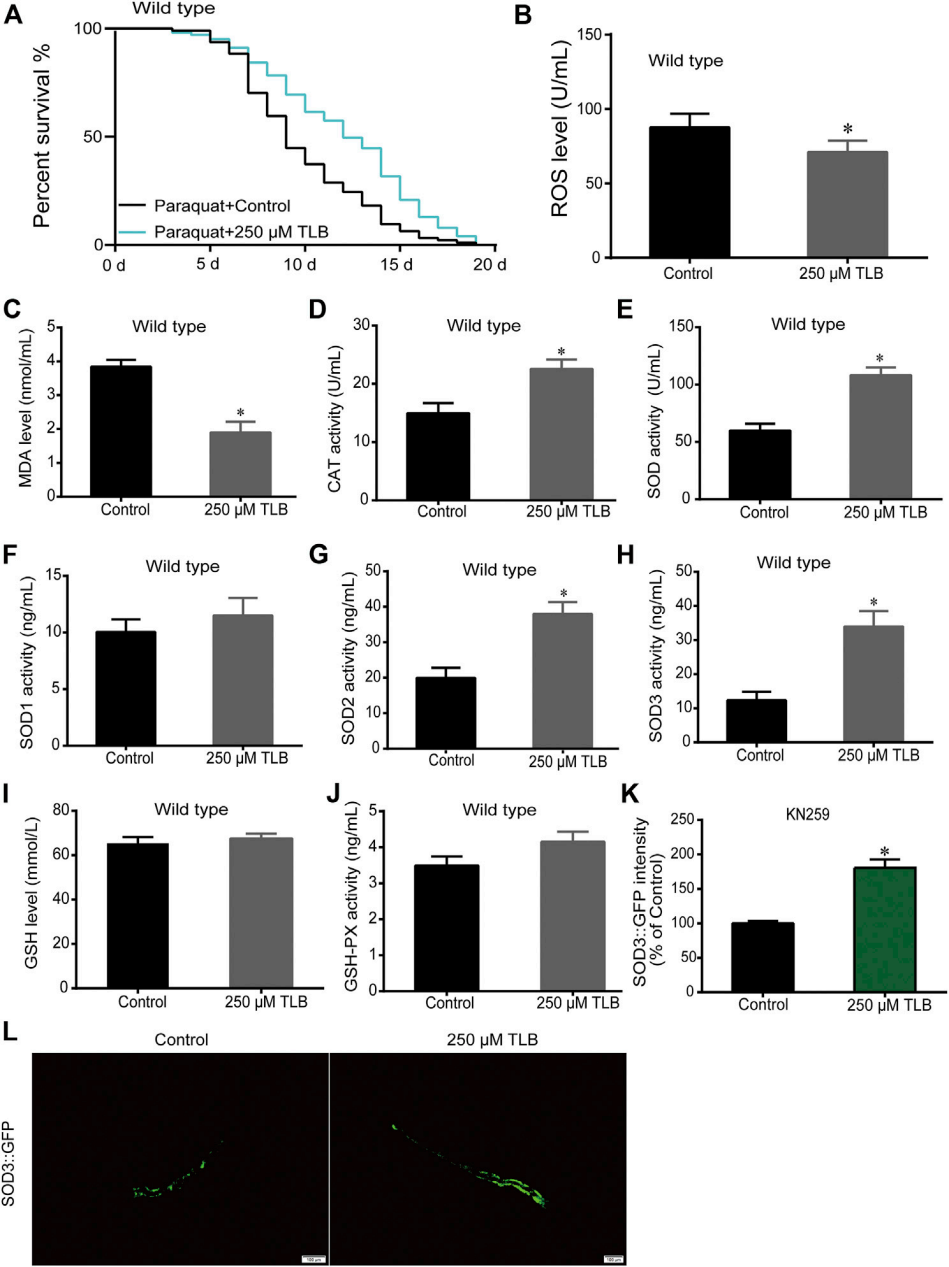
TLB reinforced the oxidative stress tolerance of *C. elegans*. Wild type *C. elegans* were treated with or without TLB after paraquat insult. **(A)** The Kaplan-Meier survival curves after paraquat insult. Wild type *C. elegans* were treated with TLB (*n* = 93–101), and *p* values were calculated by the log-rank test. *C. elegans* were raised in the absence or presence of 250 μM TLB for 72 h. The total protein of *C. elegans* was extracted and centrifuged for 10 min at 10,000 × g, 4°C, then the supernatant was detected using appropriate ELISA kits according to the manufacturer’s protocol.**(B)** ROS level (*n* = 1000); **(C)** MDA level (*n* = 1000); **(D)** CAT activity (*n* = 1000); **(E)** SOD activity (*n* = 1000); **(F)** SOD1 activity (*n* = 1000); **(G)** SOD2 activity (*n* = 1000); **(H)** SOD3 activity (*n* = 1000); **(I)** GSH level (*n* = 1000); **(J)** GSH-Px activity (*n* = 1000). **(K)** The SOD3 transgenic *C. elegans* were raised in the absence or presence of 250 μM TLB at 20°C for 72 h, and the fluorescence of *C. elegans* were observed under a fluorescence microscopy. **(L)**The mean pixel fluorescence intensity of protein SOD3 were quantified using the Image Pro Plus 6.0 software (*n* = 20). Magnification × 100; scale bar, 100 μm. Data were presented as mean ± SEM. ^*^ *p* < 0.05 vs. non-TLB treated controls.

**FIGURE 5:**
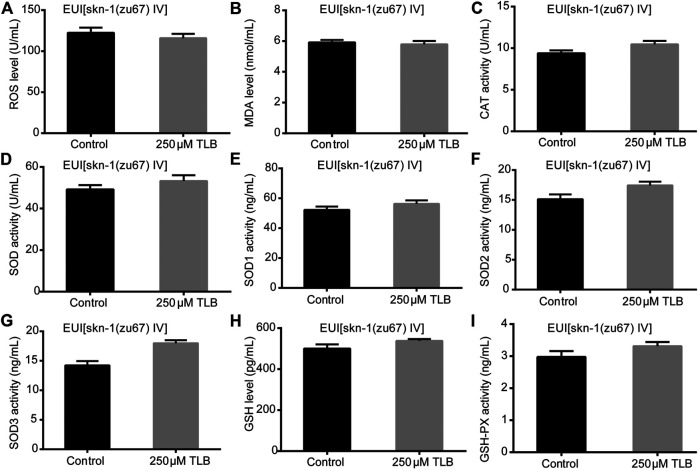
Anti-oxidant effects of TLB in SKN1 mutant *C. elegans*. *C. elegans* were raised in the absence or presence of 250 μM TLB for 72 h. The total protein of *C. elegans* was extracted and centrifuged for 10 min at 10,000 × g, 4°C, then the supernatant was detected using appropriate ELISA kits according to the manufacturer’s protocol. **(A)** ROS level; **(B)** MDA level; **(C)** CAT activity; **(D)** SOD activity; **(E)** SOD1 activity; **(F)** SOD2 activity; **(G)** SOD3 activity; **(H)** GSH activity; **(I)** GSH-Px activity. *n* = 1000. Data were presented as mean ± SEM.

**FIGURE 6:**
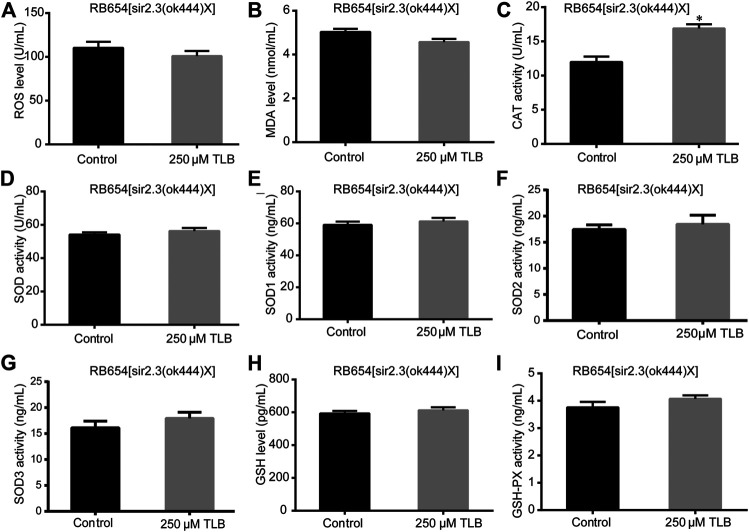
Anti-oxidant effects of TLB in Sir2.3 mutant *C. elegans*. *C. elegans* were raised in the absence or presence of 250 μM TLB for 72 h. The total protein of *C. elegans* was extracted and centrifuged for 10 min at 10,000 × g, 4°C, then the supernatant was detected using appropriate ELISA kits. **(A)** ROS level; **(B)** MDA level; **(C)** CAT activity; **(D)** SOD activity; **(E)** SOD1 activity; **(F)** SOD2 activity; **(G)** SOD3 activity; **(H)** GSH activity; **(I)** GSH-Px activity. *n* = 1000. Data were presented as mean ± SEM. ^*^ *p* < 0.05 vs. non-TLB treated controls.

**FIGURE 7:**
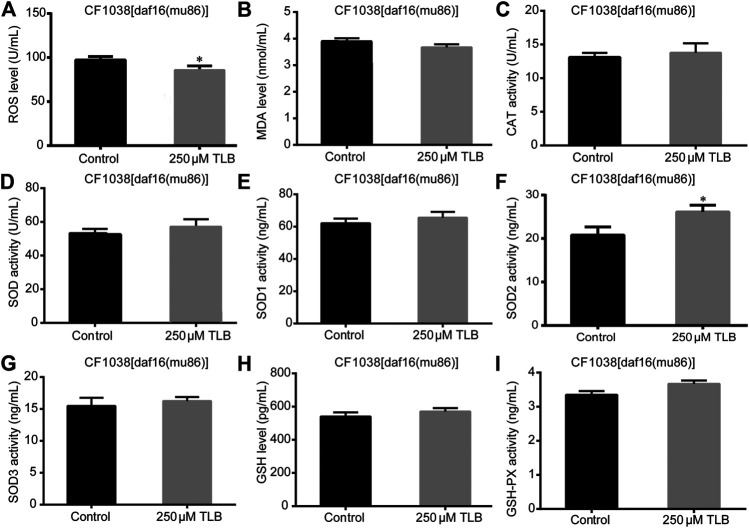
Anti-oxidant effects of TLB DAF16 mutant ***C*** *. elegans*. ***C*** *. elegans* were raised in the absence or presence of 250 μM TLB for 72 h. The total protein of *C. elegans* was extracted and centrifuged for 10 min at 10,000 × g, 4°C, then the supernatant was detected using appropriate ELISA kits. **(A)** ROS level; **(B)** MDA level; **(C)** CAT activity; **(D)** SOD activity; **(E)** SOD1 activity; **(F)** SOD2 activity; **(G)** SOD3 activity; **(H)** GSH activity; **(I)** GSH-Px activity. *n* = 1000. Data were presented as mean ± SEM. ^*^ *p* < 0.05 vs. non-TLB treated controls.

The results showed that TLB increased the lifespan than that of non-TLB treated controls after exposed to paraquat (Figure 4A). Furthermore, in order to explore the antioxidative effects of TLB, the levels of ROS and MDA, the antioxidative enzyme activities (GSH, GSH-Px, SOD, CAT) were determined. The results demonstrated that TLB decreased the levels of ROS and MDA than that of non-TLB treated controls after exposed to paraquat (Figures 4B,C). Additionally, TLB markedly strengthened CAT and SOD activities (*p* < 0.001) (Figures 4D,E). However, TLB did not affect GSH and GSH-Px activities (Figures 4I,J), while TLB significantly enhanced SOD2 and SOD3 activities, but not affected SOD1 activity (Figures 4F–H). In addition, the level of SOD3 was also quantified through utilizing transgenic strain KN259 (SOD3:GFP). The results showed that TLB significantly increased the GFP intensity of *C. elegans* than that of non-TLB controls (Figures 4K,L), indicating that TLB may be an activator of SOD, which could enhance the antioxidant ability. Whereas, the antioxidative effects of TLB were almost abolished in SKN1 (Figure 5), SIR2.3 (Figure 6) and DAF16 (Figure 7) mutant *C. elegans*. These findings suggested that longevity-enhancing effect of TLB on *C. elegans*, at least partly, through its anti-oxidant activities, indicating that TLB exerted a protective role on oxdative stress.

### DAF16/SIRT3/SKN1 Signaling Pathway Was Involved in Longevity-Enhancing Effects of TLB in *C. elegans*

**FIGURE 8:**
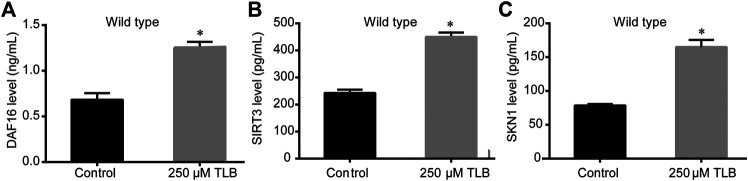
TLB increased the protein expressions of DAF16, SIRT3 and SKN1of WT *C. elegans*. *C. elegans* were raised in the absence or presence of 250 μM TLB for 72 h. The total protein of *C. elegans* was extracted and centrifuged for 10 min at 10,000 × g, at 4°C, then the supernatant was detected using appropriate ELISA kits. **(A)** DAF16 level **(B)** SIRT3 level **(C)** SKN1 level. (*n* = 1000). Data were presented as mean ± SEM. ^*^ *p* < 0.05 vs. non-TLB treated controls.

**FIGURE 9:**
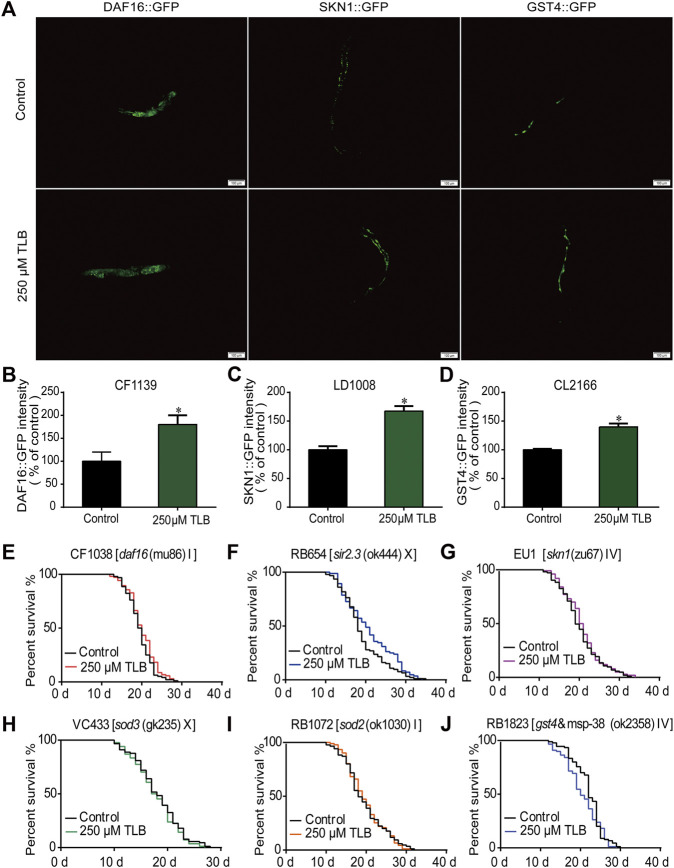
TLB extended lifespan *via* SKN1/SIRT3/DAF16 signaling pathway. Wild type or mutant type *C. elegans* were treated with or without 250 μM TLB for 72 h. **(A)** The mean pixel fluorescence intensity of proteins GST4, SKN1, and the nuclear localization of DAF16 (*n* = 20). The fluorescence of transgenic ***C*** *. elegans* were quantified using the Image Pro Plus 6.0 software. Magnification, 100 × scale bar, 100 μm **(B–D)** The quantization of levels of DAF16, SKN1 and GST4 (n = 20). All age-synchronized strains were transferred to NGM plates with or without 250 μM TLB at 20°C. Survival curves of **(E)** *daf16* (mu86) (*n* = 96–104), **(F)** *sir2.3* (ok444) X (*n* = 84–93), **(G)** *skn1* (zu67) IV (*n* = 100–103), **(H)** *sod3* (gk235) X (*n* = 83–89), **(I)** *sod2* (ok1030) (*n* = 85–91), **(J)** *gst4*&msp-38 (ok2358) IV (*n* = 88–90). Data were presented as mean ± SEM. ^*^ *p* < 0.05 vs. non-TLB treated controls.

The results of ELISA assay further demonstrated that TLB increased the expressions of the nuclear localization of DAF16, SIRT3 and SKN1(Figures 8A–C), as well as, augmented the fluorescence intensity of DAF16:GFP, SKN1:GFP, GST4:GFP than those of non-TLB treated controls (Figures 9A–D) as evidenced by the fluorescence of GFP mutants. Furthermore, the longevity-enhancing effects of TLB were almost abolished in DAF16, Sir2.3, SKN1, SOD3, SOD2, and GST4 mutant *C. elegans* than those of WT *C. elegans* (Figures 9E–J).

## Discussion

**FIGURE 10:**
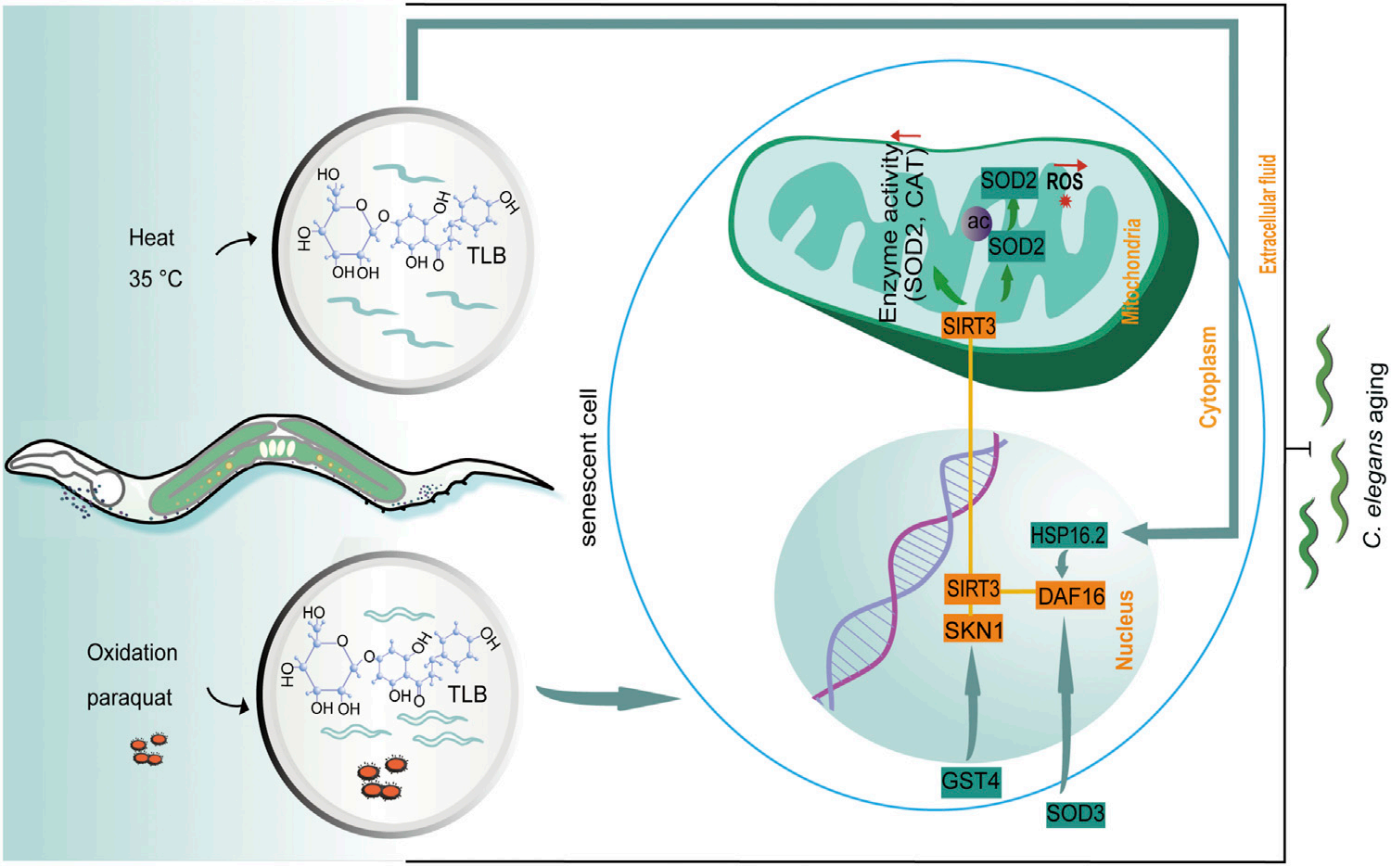
The schematic presentation illuminating proposed mechanisms that TLB prolonged lifespan in *C. elegans*. Excessive ROS production is caused by paraquat and 35°C leading to aging in *C. elegans*. TLB attenuates overproduction of ROS and improves the anti-oxidative ability through mediating the SKN1/SIRT3/DAF16 axis.

The dominating findings of this study revealed that: 1) TLB hindered natural aging of *C. elegans*; 2) TLB not only effectually reduced production of ROS and MDA, but also improved antioxidant enzymes and thermal tolerance in *C. elegans*; 3) TLB prolonged longevity of *C. elegans*, at least partly, through activating DAF16/SIRT3/SKN1 signaling pathway (Figure 10).

Accumulating evidence suggests that heat shock and oxidative stress are vital reasons of aging (Wang et al., 2016; Huang et al., 2017), which is a dominating dangerous factor for multitudinous diseases, including cancer, diabetes, multiple neurodegenerative disorders, and eventually, these changes result in living organism gradually potential to die (Edifizi and Schumacher, 2017). The senility course in *C. elegans* is rather conserved and displays resemblances to humans in many aspects such as movement, memorial capacities, immunologic function, generative rate, and cumulation of detrimental metabolites like lipofuscin. Especially, due to its characteristics of short lifespan, morphological simplicity, undemanding maintenance, and genetic manipulation, *C. elegans* was widely used to the effects of compound on lifespan (Goh et al., 2018). Our findings showed that TLB markedly prolonged the lifespan of *C. elegans* under normal or stress environment, which suggested that TLB effectively prolonged lifespan. Notably, lifespan is widely considered closely related with reproduction, the locomotion rate, food intake and lipofuscin accumulation in multiple organisms containing *C. elegan* (Wan et al., 2017; Seo et al., 2018). Our results further revealed that TLB augmented body movement rate of *C. elegans*, but it had no effect on reproduction. Since dietary restriction with reduced food intake delays aging and increases lifespan in *C. elegans*. Of note, TLB significantly decreased food intake rate, which might be contribute to the anti-aging effect of TLB.

Interestingly, TLB also mitigated the level of lipofuscin, an indicator of aging in numerous organisms (Evangelou and Gorgoulis, 2017), which further confirmed that TLB exerted longevity-enhancing effects in *C. elegans*. Of note, HSPs are a class of heat stress proteins with highly conserved structure and function. HSP generated to help proteins fold into a natural conformation under stress conditions such as high temperature, thus protected the organism and prolonged lifespan. Among them, HSP16.2 is considered as a sensitive anti-stress marker and acted as a crucial role in organism to assist correctly proteins fold (Jattujan et al., 2018; Ungvari et al., 2019). The results suggested that TLB markedly increased the HSP16.2 level and its downstream SOD3, suggesting that the longevity-enhancing effect of TLB were, at least partly, due to its anti-stress response. Furthermore, paraquat was used to generate excessive ROS and MDA to mimic oxidative damage (Moldogazieva et al., 2019), and the results demonstrated that TLB significantly decreased the levels of ROS and MDA. Moreover, it is reported that SOD has an activity of transforming intracellular superoxide anion into hydrogen peroxide (H_2_O_2_), and GSH-Px is responsible for scavenging the formed H_2_O_2_. CAT could effectively convert H_2_O_2_ to H_2_O and O_2_ (Wang D. et al., 2018). Our findings showed that TLB apparently heightened antioxidant enzyme activities of SOD and CAT, but not affected the GSH level and the GSH-Px activity, which indicated that TLB might promote the elimination of superoxide anion and H_2_O_2_ through mediating SOD and CAT activities. Of note, the SOD family is composed of three isotypes: SOD1, SOD2, and SOD3. SOD1, SOD2, and SOD3 catalyze the superoxide anion conversion to H_2_O_2_. Among them, SOD1 is mainly located in cytosolic, SOD2 and SOD3 are located in mitochondrial matrix and extracellular space, respectively (Wert et al., 2018). Notably, previous study has reported that both SOD1 and SOD2 effectively inhibit oxidative stress and SOD3 more likely contributes to prolong lifespan. Moreover, SOD2 and SOD3 are also involved in a ROS signal that functions in intercellular crosstalk and mediates longevity (Parascandolo and Laukkanen, 2019). Our results showed that TLB significantly enhanced SOD2 and SOD3 activities, but not affected SOD1 activity, which suggested that TLB might mainly eliminated the ROS of mitochondrial matrix and extracellular space of cells, but not the ROS of cytosolic. These findings suggested that the anti-oxidative effect of TLB mainly due to eliminating excessive ROS in mitochondria and promoting the lifespan-related SOD2 and SOD3 in *C. elegans*.

Emerging evidence suggests that SOD2 is the downstream of SIRT3, which is located in mitochondria and attenuated mitochondrial ROS (Katwal et al., 2018). In our previous study, a major upstream of SIRT3 that has been proved is NRF2, which is encoded as SKN1 gene on *C. elegans* (Gao et al., 2018; D'Amora et al., 2018). Our results demonstrated that TLB upregulated the protein expressions of SKN1 and SIRT3, thereby exerting its anti-oxidantive effect; whereas, the anti-oxidantive effect of TLB were almost eliminated in SKN1 and SIRT3 mutant *C. elegans*, which indicated that NRF2/SIRT3 signaling pathway was involved in the anti-aging effects of TLB in *C. elegans*. In addition, SOD3 is known as a downstream gene of DAF16, which is an ortholog of the FOXO gene in *C. elegans* (Wang et al., 2017). Our results showed that TLB significantly increased DAF16 level, and the beneficial effect almost was abolished in DAF16 mutant *C. elegans*. Since FOXO3 is the downstream of SIRT3(Wang L.-F. et al., 2018), thus it is reasonably assumed that SKN1/SIRT3/DAF16 signaling was involved in the longevity-enhancing effect of TLB in *C. elegans*; whereas, the mechanistic details of how TLB effect the DAF16, SIRT3, and SKN1 expression will be further explored in our next story.

Of note, emerging evidence reveals that there are close relationship between polyphenol compounds, TCM, redox status and the vitagene network, which encodes for heat shock proteins, the thioredoxin and the sirtuin protein systems, and its possible biological relevance in neuroprotection (Wang Y. et al., 2018). Moreover, preconditioning signal leading to cellular protection through hormesis, terms as a dose response phenomenon characterized by a low dose stimulation and a high dose inhibition, is an important redox dependent aging-associated neurodegenerative/neuroprotective issue (Calabrese et al., 2010; Calabrese et al., 2018; Brunetti et al., 2020; Rosa et al., 2020; Siracusa et al., 2020). We speculated that TLB might be a promising natural radical scavenger for the prophylaxis and neuroprotective effects, thereby exerting an anti-aging effect. To this regard, the pharmacokinetic and pharmacodynamics functions and hormetic properties of TLB in other aging animal models such as SAMP8 mice will be further elucidated.

Taken together, the present study demonstrates that, for the first time, TLB effectively prolongs lifespan of *C. elegans*, at least partially *via* modulating SKN1/SIRT3/DAF16 signaling pathway. These findings raise the possibility that TLB may be a potential and effective agent against aging or aging-related diseases.

## Data Availability

The original contributions presented in the study are included in the article/Supplementary Material, further inquiries can be directed to the corresponding author.
